# Approaches to Biofunctionalize Polyetheretherketone for Antibacterial: A Review

**DOI:** 10.3389/fbioe.2022.895288

**Published:** 2022-05-13

**Authors:** Yihan Wang, Shutao Zhang, Bin’en Nie, Xinhua Qu, Bing Yue

**Affiliations:** ^1^ Department of Bone and Joint Surgery, Renji Hospital, School of Medicine, Shanghai Jiaotong University, Shanghai, China; ^2^ Department of Orthopedics, Renji Hospital, School of Medicine, Shanghai Jiaotong University, Shanghai, China

**Keywords:** PEEK, antibacterial properties, modification technology, polyetheretherketone, implant materials

## Abstract

Due to excellent mechanical properties and similar elastic modulus compared with human cortical bone, polyetheretherketone (PEEK) has become one of the most promising orthopedic implant materials. However, implant-associated infections (IAIs) remain a challenging issue since PEEK is bio-inert. In order to fabricate an antibacterial bio-functional surface, modifications of PEEK had been widely investigated. This review summarizes the modification strategies to biofunctionalize PEEK for antibacterial. We will begin with reviewing different approaches, such as surface-coating modifications and controlled release of antimicrobials. Furthermore, blending modifications and 3D printing technology were discussed. Finally, we compare the effects among different approaches. We aimed to provide an in-depth understanding of the antibacterial modification and optimize the design of the PEEK orthopedic implant.

## Introduction

The ideal orthopedic implant should possess good histocompatibility, good mechanical properties close to bone tissue, good bone–implant integration, and adequate antibacterial property (Liu et al., 2019). Currently, titanium is widely used in orthopedic clinical implants, followed by stainless steel, Mg, and other metals (Kim et al., 2020; Yang et al., 2020). However, the stress shielding effect of metal implants cannot be solved so far, which eventually leads to bone loss and loosening of implants (Heary et al., 2017; Mishchenko et al., 2020). Therefore, it is of great clinical and scientific significance to develop novel orthopedic implant materials. Among these, polyetheretherketone (PEEK) is a widely explored alternative orthopedic implant material. Since it was approved as a medical implant material by the FDA in the late 1990s, many researchers have paid attention to its application prospects in the field of orthopedic (Panayotov et al., 2016).

**FIGURE 1:**
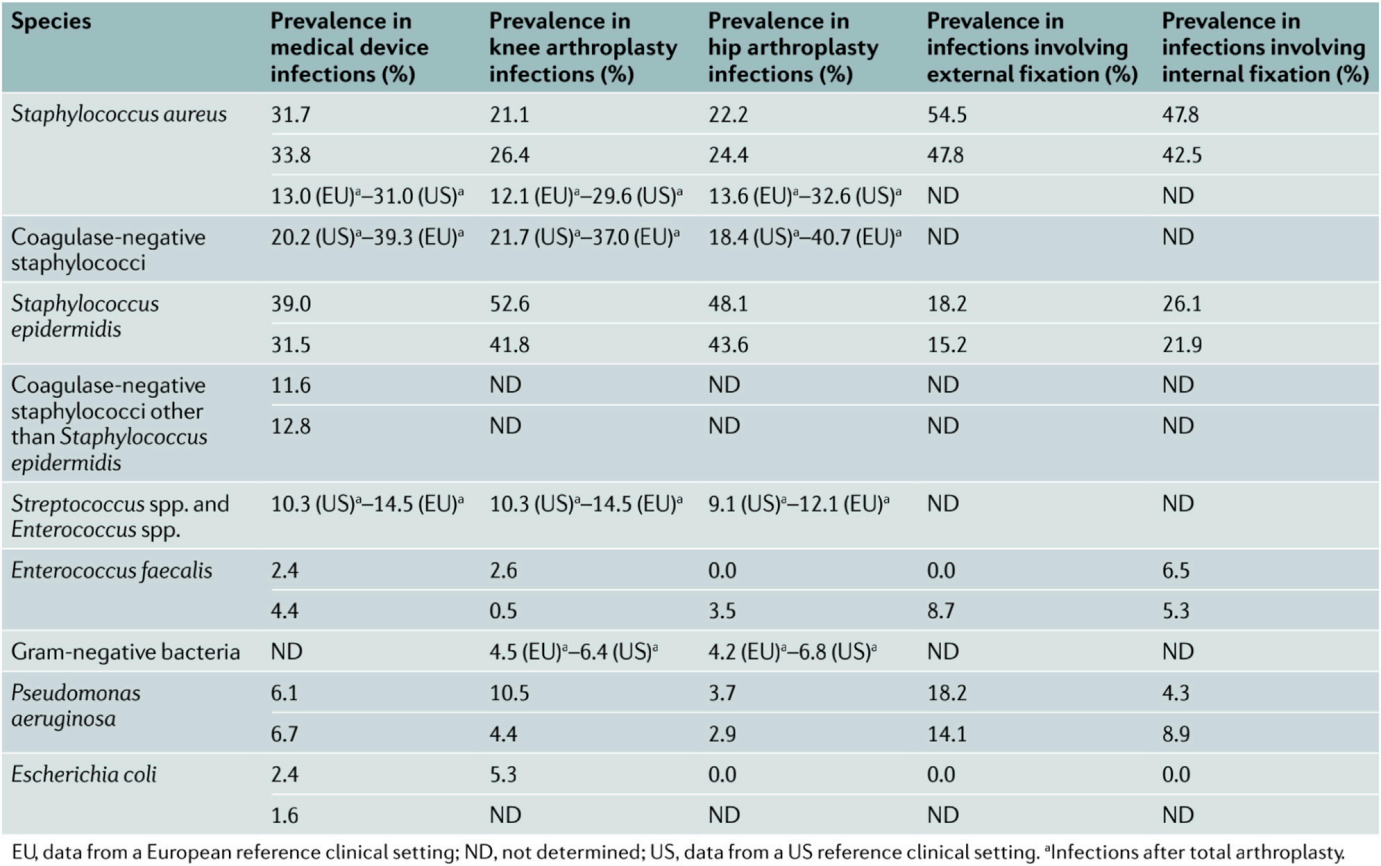
Major bacteria causing implant-associated infections. Reproduced from Arciola et al (2018). Copyright (2018), with permission from Springer Nature.

The average rate of orthopedic implant-associated infections (IAIs) is about 2–5%, even up to 30% of open fractures (Kuehl et al., 2019), which bring serious physical and psychological trauma to patients and generate a lot of medical expenses (Wang and Tang, 2019). Hip and knee arthroplasty has been a very mature technology in the treatment of terminal arthritis. Periprosthetic joint infections (PJIs) are the most serious complication, although the incidence is not high; it can be divided into acute and chronic infection based on the choice of clinical treatment (Otto-Lambertz et al., 2017). Whether the biofilm formatted on the surface of the prosthesis is one of the important factors determining acute or chronic infection needs further investigation. Compared with the treatment of acute infection by antibiotics, the treatment of chronic infection is more difficult. Two-stage revision is still the gold standard at present (Matar et al., 2021). PJI can lead to prosthesis loosening and dislocation, etc., and even if the infection is controlled by treatment, the long-term reoperation rate of joint revision is higher than that of uninfected patients (Clauss et al., 2020). In IAI, the most commonly cultured pathogens are Gram-positive; many other microorganisms can also be found (Figure 1). One of the reasons for the poor prognosis of IAI is failing to identify the pathogen species of infection at the first time. In addition, granulation tissue generated around the implant forms the immune depression area, which is prone to infection and colonization (Arciola et al., 2018). This is due to biofilm formation and then diffuses infection of surrounding tissues resulting in poor response to antibiotic therapy and may eventually lead to loosening or even shedding of the implants (Naik et al., 2015). Single-celled bacteria form biofilm through four stages: bacterial surface attachment, small colony formation, bacterial biofilm maturation, and dispersion separation (Bjarnsholt, 2013). The bacteria in the multilayer form of biofilm are in a static or dormant period, so they can resist clinical antibiotic treatment and phagocytes of the immune system (Zimmerli and Sendi, 2017; Masters et al., 2019). Therefore, the formation of bacterial biofilm will seriously affect the role of antibiotics and make treatment difficult. Until now, the incidence rate of IAI has not declined significantly even with strict aseptic techniques and application of broad-spectrum antibiotics (Wang and Tang, 2019). The modifications of implant materials to inhibit the initial bacterial adhesion and the formation of biofilms have great prospects in reducing the rate of IAI. Seeking new antibacterial implant materials is another way (Jiao et al., 2021), which requires abundant research resources. As new material needs to meet the requirements of biomechanics, histocompatibility, and osseointegration, meanwhile, it needs stable chemical properties, easy plasticity, low cost, and convenience for mass production. Consequently, surface biofunctionalization including chemical and physical modifications by antibacterial nanoparticles or compounds of existing approved materials to achieve antibacterial properties is a more efficient approach.

PEEK is an ideal bone substitute material due to its bio-inert property, safety, non-toxicity, wear-resistance, transmit radiation, strong plasticity, and mechanical properties close to the human cortical bone (Ya-Shum et al., 2018; Ma Z. et al., 2020; He et al., 2021). It had been successfully used as a biomaterial for arthroplasty construction and trauma and dental implant applications (Table 1). However, it also has drawbacks, for example, the unique structure leads to surface hydrophobicity that inhibits protein and cell adhesion (Ji et al., 2020), resulting in poor osseointegration and failure of the implant (Liao et al., 2020). In addition, it is easier to colonize bacteria and form biofilms due to its bio-inert property (Bock et al., 2017). Therefore, to make PEEK more suitable for orthopedic implant materials, it must be biofunctionalized to improve biocompatibility, osseointegration, and antibacterial properties. In recent years, the development of biomaterial modification has been greatly promoted relying on the progress of nanotechnology (Hochella et al., 2019). Modified biomaterials, such as nano-hydroxyapatite, have shown improved osseointegration over micro-hydroxyapatite (Panayotov et al., 2016; Lowe et al., 2020). PEEK also can get better modification effects and less inflammatory reaction due to nanotechnology (Gu et al., 2020). Nowadays, a large number of research studies have been carried out successfully *in vitro* and *in vivo* (Torstrick et al., 2018; Chepurnyi et al., 2020; Liu et al., 2020), but there is still a lack of in-depth understanding of the modification of antibacterial properties.

**TABLE 1:** Clinical application of PEEK as implant.

Material	Model	Application	Patient	Observation	Outcome	Reference
Porous PEEK	3D-printed CAD models	Sub-periosteal implants in buccal cavity	5 patients	Follow-up for 12 months	All implants were not showing any signs of mobility, infection, or prosthetic fracture	Mounir et al. (2018)
PEEK-on-highly cross-linked polyethylene	Injection-molded	Joint prosthesis for TKA	10 patients (9 females and 1 male) with mean age 66.9 years	Imaging evaluations (10 cases at 1 and 3 months; 7 cases at 6 months)	The condition of periprosthetic bone volume and prosthesis position can be well assessed	Cai et al. (2022)
PEEK cage	—	Lumbar fusion with the interbody cage	1,094 patients (673 patients using a PEEK cage, while others using a Ti cage)	Meta-analysis from 11 studies	PEEK cage associated with lower odds of leg pain but lower fusion rate; no difference in subsidence rates and (VAS)-low back pain	Massaad et al. (2020)
Pure PEEK	3D-printed CAD model	Scapular prosthesis after tumor resection	A 16-year-old male	Follow-up at 1 month and 3 months	Constant shoulder score (CSS) was 68 points; the X-ray of the normal position of the shoulder joint; and no complications	Liu et al. (2018)
Pure PEEK	3D printed	Clavicle prosthesis after excision due to osteomyelitis	A 23-year-old female	Follow-up for 2 years	Pain VAS was 0/10 at 3 months; CSS was satisfactory with 88 points; no implant loosening or other complications during 2 years	Chang Chen et al. (2021)

The clinical application of PEEK implants has continuously reported cases of infection (Roman et al., 2014; Chen et al., 2019; Tarallo et al., 2020; Hacherl et al., 2021). In this review, we focused on the antibacterial modification strategies of PEEK. Meanwhile, the biocompatibility and osseointegration are also taken into consideration on that the rapid integration of implant and surrounding tissue being crucial for preventing bacterial adhesion and colonization (Stones and Krachler, 2016). Because the implants cannot be absolutely sterile due to exposure to the air during the operation and there is no guarantee of any microbial colonization in the body for decades, only antibacterial modifications of the implant surface are futile. The formation of bacterial biofilm can be regarded as the competition of adhesion between bacteria and surrounding tissue cells (Subbiahdoss et al., 2009; Busscher et al., 2012), which was called “race for the surface,” as first proposed by Gristina (1987). Ultimately, the objective of this article was to discuss the advantages and disadvantages of different modifications hoping to inspire and reference future research in this field.

### Current Research on Biofunctionalized PEEK With Antibacterial

**FIGURE 2:**
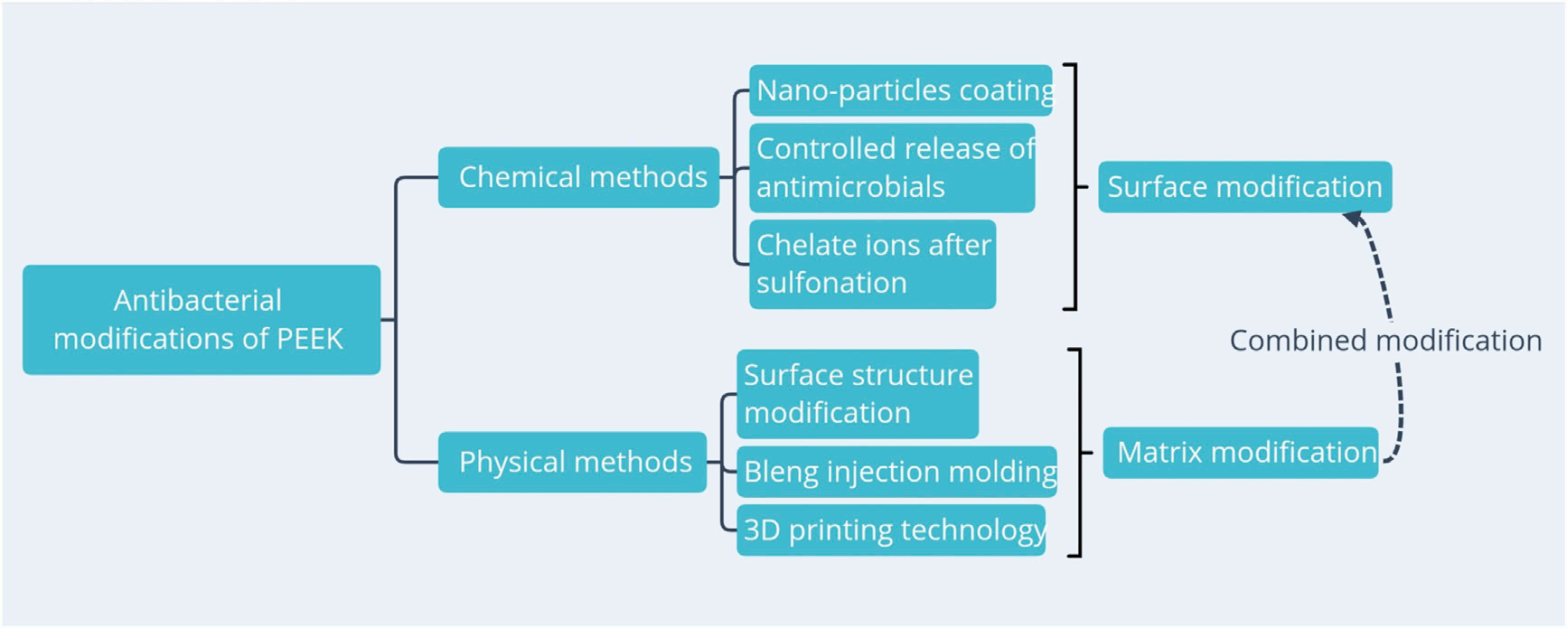
Current research on biofunctionalized PEEK with antibacterial.

At present, there are many approaches to biofunctionalize PEEK for antibacterial, and fruitful results have been achieved (Poulsson et al., 2019). According to the biofunctionalization approaches as well as 3D printing technology, it can be divided into chemical modification and physical modification (Figure 2). Chemical modification is to functionalize the PEEK with antimicrobial particles or antimicrobial compounds. Physical modifications include changing the PEEK surface structure and particle blending and injection molding to form composites to meet application requirements. The authors screened the parts involving antibacterial properties in PEEK modification research in recent years, summarized, and discussed them hierarchically.

### Chemical Modification

#### Nanoparticle Coating

The coating technique is the earliest and most widely used in PEEK surface modifications, such as magnetron sputtering (Liu et al., 2017), plasma treatment (Zhang Y. et al., 2021), and bioglass immobilization (Huo et al., 2021b). The advantage is that it can directly improve the biocompatibility, osseointegration, and antimicrobial properties of the PEEK, without destroying the mechanical properties of its matrix. However, there are drawbacks, such as the risk of coating peeling, which may lead to bone resorption and implant failure (Niu et al., 2020), and the cytotoxic risk of metal particles (AshaRani et al., 2009). To minimize the excessive absorption of metal particles, improvements can be made in terms of enhancing the firmness of the coating and the controlled release of the minimum effective concentration of metal ions. At present, there are two main ideas. One is to make nano-metal particle composites to enhance their adhesion. It is reported in the literature that silver nanocluster–silica composite was sprayed on the surface of PEEK composites by radio frequency sputtering. It was proved that it retained good antibacterial properties by bacterial experiments *in vitro*, while improving the stability of the coating, and silica regulating the release of silver ions, thereby reducing cytotoxicity (Ur Rehman et al., 2017). Another approach is adding a controlled release coating on the particles to achieve the purpose of stabilizing the metal nanoparticles and controlling the release of metal ions. J. Kratochvíl and other scholars deposited copper nanoparticles on the surface of PEEK and further coated it with the fluorocarbon plasma polymer (C: F) film. The experimental results showed that when the thickness of the composite coating was 10 nm, it not only increased the coating stability and controlled release but also retained the antibacterial properties of copper ion and hindered the formation of biofilms (Kratochvíl et al., 2018). The same approach can be applied to silver nanoparticle coatings (Kylian et al., 2017). However, considering that orthopedic implants often exist in the human body for several years or even decades, no matter how low the cytotoxicity is in the short term, the risk of long-term excessive release and absorption of metals must be considered and verified. There is currently a lack of long-term *in vivo* trials of surface-modified PEEK coated with nano-metallic particles, thus lacking strong evidence for an in-depth discussion of its safety and antibacterial efficacy.

To avoid the cytotoxicity of metal particles, some researchers have explored non-metallic nanoparticles with antibacterial activity. Wang et al (2016) prepared red selenium and gray selenium nanocoatings on the surface of PEEK and found that short-term (<3 days) inhibited *Pseudomonas aeruginosa*, which could reduce infection and inhibit the formation of biofilms. Compared with nano-Ag particles, selenium nano-ions have been proven to be healthier, safer, and less cytotoxic (Perla and Webster, 2005). Buwalda et al (2020) immobilized ethylene glycol and dimethylamino ethyl acrylate on the surface of PEEK by UV radiation, and *in vitro* experiments demonstrated that it had a good inhibitory effect on *E. coli* and *S. aureus*. Non-metallic coatings also need to consider the antibacterial efficiency, such as the antimicrobial peptide of GL13K and 1-ethyl-3-(3-dimethyl aminopropyl) carbodiimide-mixed coating, of which 1-ethyl-3-(3-dimethyl aminopropyl) carbodiimide-mixed coating exhibited better antimicrobial performance than pure GL13K coating (Hu et al., 2021). Our group also carried out some work on biofunctionalized PEEK. We found that the PEEK surface modified with hydrofluoric acid and nitric acid (AFN) could promote M2 polarization of the macrophages and stimulate the differentiation of osteoblasts (Huo et al., 2021a).

**FIGURE 3:**
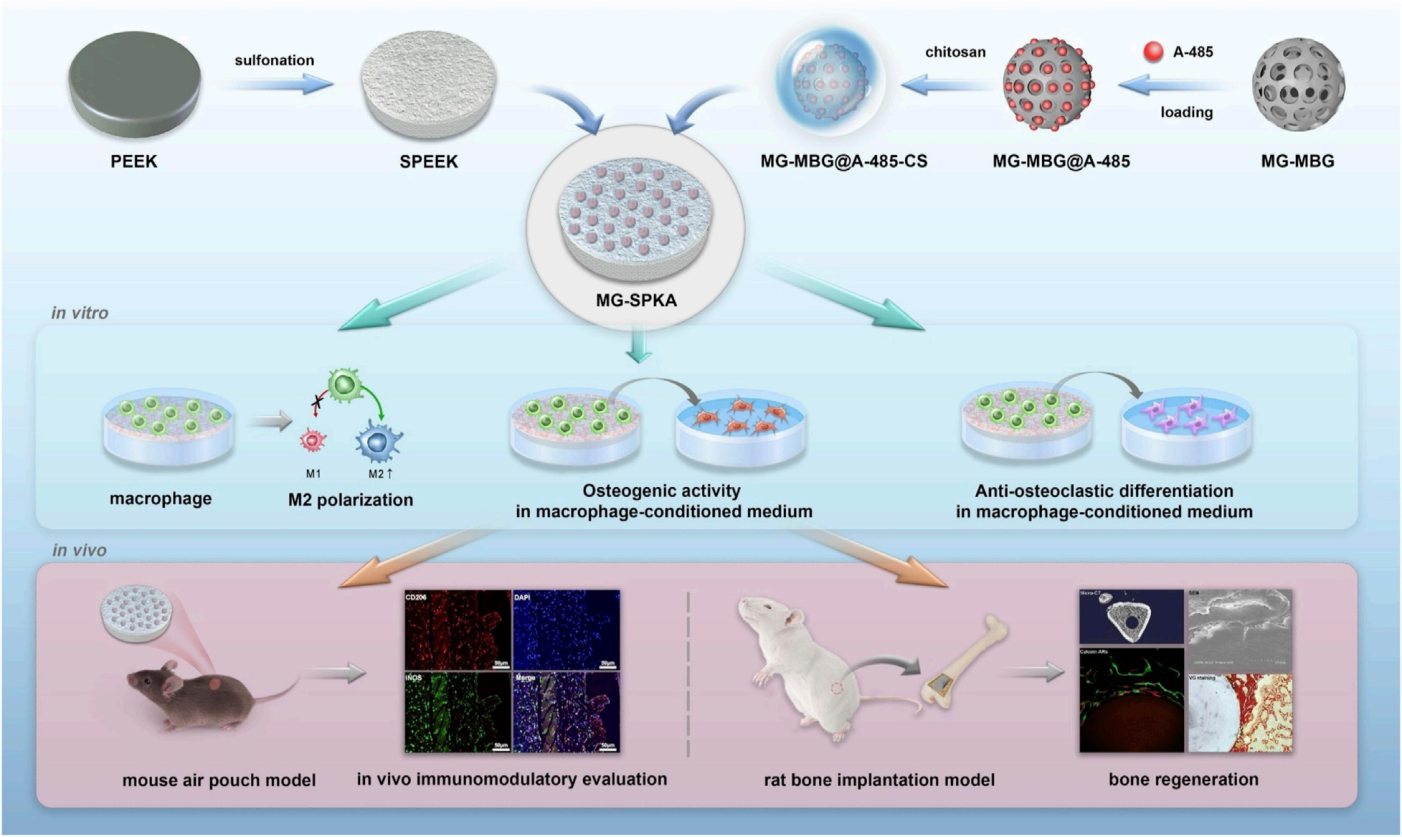
Schematic diagram of the process used to prepare and evaluate the PEEK materials. Reproduced from Huo et al (2021c). Copyright (2021), with permission from Elsevier.

In addition to that, PEEK was biofunctionalized with mesoporous bioactive glass (MBG) and loaded with A-485 for attenuating inappropriately activated immune responses and inhibiting osteoclastogenesis, thus promoting osteogenesis (Figure 3). Also, this MBG could also perform as long-lasting antibacterial agents for antibacterial. The advantage of PEEK surface coating modification is that the approach is simple and conducive to large-scale production, but the release of functional particles, the cytotoxicity of exfoliated particles, and the tissue inflammatory response still require in-depth research and long-term observation in the animal *in vivo* experiments.

### Controlled Release of Antimicrobial Agents

The most common modification for antibacterial compounds or drugs is further polymerization on porous PEEK surfaces by polydopamine (PDA) coating. Polydopamine is inspired by mussels foot proteins and reported by Lee et al (2007) and has rapidly become a widely used method for surface decoration of materials, which can form functional nanocoatings on almost all material surfaces and can further polymerize other compounds (El Yakhlifi and Ball, 2020). Moreover, PDA coating exhibits synergistic antibacterial properties and can be used for photothermal therapy (Niyonshuti et al., 2020; Singh et al., 2020); thus, it has enormous potential in the biomedical field. The PEEK-PDA surface after antibacterial modification has also been proved with good biocompatibility and osseointegration (Table 2).

**TABLE 2:** Controlled release of antimicrobial agents on the PEEK-PDA (2 mg/ml dopamine hydrochloride) surface.

Ion	Sample	Method	Bacterial strain	Antibacterial test	Antibacterial property	Mechanism	Biological effect	Reference
AgNPs	Tollens’ reagent (0.02 mol/L) [ammonia into AgNO_3_ solution (0.02 mol/L)]	PEEK-PDA immersed in the Tollens’ reagent for 30 min	*E. coli* , S. *aureus*, and S. *aureus*	Plate counting and SEM IAI model in rat femur	97.9 ± 0.8% 99.8 ± 0.1% (antibacterial rate) *S. aureus* growth was significantly inhibited on the surface	Fast Ag+ release initially, then slow release for long-term	Showed low toxicity to MC3T3-E1 cell and possessed good biocompatibility and osseointegration	Gao et al. (2017)
AgNPs、SF/GS	AgNO_3_–gentamicin (500 lg/mL in PBS) and silk fibroin	SP–PDA–Ag immersed into gentamicin followed by three silk layers	*E. coli* and S. *aureus*	Plate counting and SEM	Greatly enhanced the antibacterial effects and antiadhesion ability	The release rate of Ag+ and GS increased with decreasing pH	PDA and silk fibroin can balance cytocompatibility and antibacterial ability	Yan et al. (2018)
KR-12	KR-12 solution (1 mg/ml in 10 mM Tris–HCl buffer)	PEEK-PDA immersed in KR-12 solution under reverse nitrogen (N_2_) flow overnight	S. *aureus* and S. *aureus*	Plate counting and SEM IAI model in rat femur	Effectively inhibited bacteria proliferation and biofilm formation *in vitro* and *in vivo*	Release of KR-12 which has a broad spectrum of antibacterial activity	rBMSCs: improved adhesion, proliferation, and osteogenic differentiation; in vivo: promoted osteointegration in rat femur	Meng et al. (2020)
GS	PDA and GS diluted in Tris–HCl solution (2 mg/ml and 3 mg/ml)	SPEEK was immersed in the left solution for 12 h	*E. coli* , S. *aureus*, and S. *aureus*	Plate counting IAI model in rat femur	Continuous antibacterial abilities. Imaging showed no evidence of osteomyelitis	GS release	Possessed good biocompatibility and the immunoregulatory ability	Sun et al. (2021)
Mino liposomes	Minocycline (Mino), liposome, and dexamethasone (DEX)	PEEK-PDA immersed in the Dex/Mino liposome solution obtained by the thin-film hydration method	S. mutans, P. gingivalis, and S. mutans	Plate counting and microbial viability assay kit-WST subcutaneous infection model of rats	Improved the antibacterial activity and inhibited the initial adhesion. The antibacterial efficiency was about 97.4%	Liposomal Mino releasing and benign cell adhesion on the functionalized PEEK surface	*In vitro*, improved osteogenic differentiation of human mesenchymal stem cells. *In vivo*, enhanced osteointegration	Xiao Xu et al. (2019)
Zn^2+^–Mg^2+^	Mg(NO_3_)_2_·6H_2_O、Zn(NO_3_)_2_·6H_2_O、2,5-dihydroxy-terephthalic acid (DHTA) dexamethasone (DEX)	PEEK-PDA was dipped in Zn−Mg-MOF74 composite obtained by hydrothermal synthesis and then coated by DEX	*E. coli*, S. *aureus*, and S. *aureus*	Plate counting and SEM fluorescence microscope subcutaneous infection model of rats	Significantly inhibited bacterial proliferation. Much smaller number of bacteria	Mg^2+^, Zn^2+^, and DHTA release. The alkaline microenvironment due to the coating degradation	Improved vascularization and osteogenic differentiation	Xiao et al. (2021)
Van-GNPs	vancomycin (Van)–Gelatin nanoparticles (GNPs)	Plasma modification (PDA/P-PEEK) combines two-step desolvation (Van-GNPs)	*S. aureus* and S. mutans	Plate counting and SEM	Inhibited bacteria adhesion and the cell membrane bursted on the Van-GNPs/PEEK surface	Vancomycin release	Good osteogenesis without cytotoxicity	Tianjie Chen et al. (2021)
AgNPs–µCuO/SF	Cu(NO_3_)_2_ •3H_2_O、AgNO3, Silk fibroin SPEEK	μCuO was prepared by hydrothermal solution followed by polymerization	*E. coli* and S. *aureus*	Plate counting and SEM	Effectively inhibited bacteria adhesion and biofilm formation	Cu^2+^ and Ag^+^ release	Showed better osteogenesis in the rabbit tibial defect model	Yan et al. (2020)

Silver nanoparticles (AgNPs) can destroy bacteria walls (Choi et al., 2008). AgNPs were reduced from [Ag(NH3)2]^+^ utilizing the reducibility of the catechol in PDA, which exhibited superior and long-term antibacterial properties against *E. coli and S. aureus. However,* there were no notable differences in not only pro-inflammatory but also anti-inflammatory gene expression between PEEK–PDA–Ag and blank groups which was significantly lower than that in the PEEK group due to the immunosuppressive effect of AgNPs (Gao et al.bgmj, 2017). In addition to antibacterial nanoparticles, integrating antibiotics are also one of the common modification approaches without worrying about the toxicity of metal ions. Sun et al (2021) prepared the SP-GS/PDA functional surface, which rapidly released gentamicin (GS) in the SBF solution. Its antibacterial activity sustained high during the initial 3 days but lost on day 4 due to the little residual content of GS. The long-term stable release of antibiotics with sub-lethal concentration is an urgent problem to be solved. However, Yan et al. (2018) explained different experimental results, and they believed that during the first day, neither AgNPs nor GS alone improved antibacterial activity significantly, yet the combination of them enhanced the antibacterial properties greatly. Thereafter, surface modification with AgNPs and copper oxide microspheres (μCuO) was explored. Yan et al (2020) proposed SP-CuO/Ag-enhanced osteogenesis while ensuring antibacterial properties. It can be seen that the effect of combined modification by multiple antibacterial particles is not a simple superposition, which needs further experimental exploration in the future. The biofunctionalized PEEK which is suitable for implant needs to not only enhance antibacterial properties but also improve histocompatibility and bone osteointegration. Some scholars have made attempts in this regard: Xu X. et al. (2019) considered that dexamethasone (DEX) was an osteoinducing and anti-inflammatory factor easing foreign body response to PEEK implants. They prepared PEEK-DEX/Mino liposome that was proved to be anti-inflammatory and osteogenic besides not reducing antibacterial properties. Modification of combined antibacterial metal nanoparticles was also explored. Compared with Zn−Mg-MOF74/PDA composite, DEX@Zn−Mg-MOF74/PDA composite coating on PEEK showed similar antibacterial properties but better osteogenic gene expressions (OPN and OCN) of rBMSCs observed by RT-PCR (Xiao et al., 2021). PDA coating has the advantage of simple operation and can integrate a variety of modification factors through interlayer. However, these experiments lack research on the wear resistance and stability of PDA coating. Chen et al. believed that the binding strength could be improved by argon plasma surface modification before PDA coating onto the PEEK (Chen et al., 2021). More similar attempts need to be verified in the future.

In addition, other coating modification techniques have also been applied: lawsone, an antibacterial compound that can be applied topically *in vivo* (Sultana et al., 2021), was made into a suspension with the bioactive glass powder and chitosan by Ur Rehman et al. (2018). It was found that lawsone maintained good antibacterial properties without affecting the biological activity of the material itself. Brushite (CaHPO4·2H2O) and gentamicin were coated on PEEK through a layer-by-layer (LBL) method with varied cycles. The antibacterial efficiency and osseointegration ability exhibited differences due to the number of cycles (Xue et al., 2020). Different modification techniques will change the release concentration and time of antimicrobial compounds. Considering that too high concentration of a local drug may affect bone healing and produce cytotoxicity and very long release time may lead to local bacterial resistance. Ideal antimicrobial compound coating modification should have broad-spectrum antibacterial, low toxicity, controlled release at minimum effective concentration, and ideal release time. In addition, some problems in this modification method to be verified are: whether the coating is stable; whether the antibacterial activity and release rate will be affected *in vivo*; as a bone implant, it will withstand different stress and friction; whether it can maintain the effective antimicrobial drug concentration per unit area. In a word, the surface coating approaches of PEEK antibacterial modification have been fruitful, but so far, most of the research is based on *in vitro* experiments, and the safety and effectiveness of *in vivo* experiments, especially under biomechanics conditions still lack long-term observation.

### Chelated Antibacterial Ions After Sulfonation of PEEK

Sulfonated PEEK (SPEEK) fabricated by concentrated sulfuric acid makes the surface of PEEK form a negatively charged (-SO3H) interface, which repels the bacterial biofilm that is also negatively charged, thereby having antibacterial properties (Tomoglu et al., 2019). In addition, it can rely on ionic bonds to further chelate antibacterial ions to improve antibacterial performance (Table 3). Compared with the multi-coating adhesion methods, this approach retains the three-dimensional micro-nanostructure of the contact interface and may have stronger coating stability because of the chelation of positive and negative ion bonds. Silver is still one of the most widely used ions, and scholars have been exploring more synergies with other particles (Sikder et al., 2018; Yu et al., 2021). For example, compared with Ag-SPEEK, Ag/Zn-SPEEK (dual Ag^+^ and Zn^2+^-decorated SPEEK) retained similar antibacterial properties but showed better cytocompatibility and osseointegration (Deng et al., 2018). In addition, the multiple assembly methods of modified ions produced different effects. Yang et al. (2022) fabricated zeolitic imidazolate framework-8 (ZIF-8) on SPEEK from 2-methylimidazole and Zn^2+^, which possessed a high Ag^+^-loading capacity. Experimental results demonstrated that SPZA had more effective antibacterial properties. The improvement of antibacterial activity was obtained not only by the synergy between different metal ions but also by the mixed modification of metal ions and antibacterial compounds. Li^+^ could enhance cell attachment, proliferation, differentiation, and implant−bone interface osseointegration of the antibacterial AMP coating on SPEEK (Li N. et al., 2021). Considering that antibiotics may lead to drug resistance and metal particles may lead to cytotoxicity, scholars pay attention to antimicrobial peptides (AMPs). However, AMPs are easily inactivated attributing to inducing autoimmune responses (Brandwein et al., 2017); consequently, an AMP from human elements is crucial to endophyte application. Recombinant mouse beta-defensin-14 (MBD-14) which has high homology as well as human beta-defensin-3 (HBD-3) was embellished on the SPEEK. The experimental result indicated that SP-MBD had good antibacterial activity, biocompatibility, and osteogenesis (Yuan et al., 2019). According to this idea, Yang et al. (2019) loaded sodium butyrate, a fermentation product of gut microbiota, onto the SPEEK surface and found that SB-SPEEK exhibited superior anti-infection capacity and induced new bone formation *in vitro and in vivo*. We can also combine AMPs and osteogenic promoters. Chlorogenic acid (CGA) has excellent antibacterial properties, and grafted peptide (BFP) is one of the most effective bone-inducing growth factors. He et al. (2019) fabricated a hydrogel of compound coating with sodium alginate (SA) on the SPEEK surface and revealed that SPEEK@SA (CGA)@BFP effectively inhibited bacterial proliferation and showed better bioaffinity. Any antibacterial modification method cannot sacrifice bone integrity and biocompatibility, and the detection of cytotoxicity is a crucial experiment before entering the clinical trial. Therefore, the research on antibacterial substances from biological sources has broad prospects.

**TABLE 3:** Sulfonated PEEK further chelates antibacterial ions.

Ion	Sample	Method	Bacterial strain	Antibacterial test	Antibacterial property	Antibacterial mechanism	Biological effect	Reference
GO	Graphene oxide powders	Dip-coating in GO–water suspension	*E. coli* and S. *aureus*	Plate counting and SEM	Effectively inhibited the proliferation of *E. coli* but failed S. *aureus*	Creates a neutralized surface state; induce oxidative stress and membrane stress	Higher ALP and osteogenic differentiation-related genes activity and more calcium nodule formation	Ouyang et al. (2018)
Ag^+^/Zn^2+^	Nacl, Alginate/Ag^+^, and Chitosan/ZnO	Layer-by-layer on SPEEK (dip-coating)	*E. coli* and S. *aureus*	Plate counting and SEM	Greatly suppressed the growth of bacteria	The nano-Ag-containing interface and chitosan in conjunction	Enhanced expression of MG-63 ALP and osteogenesis-related genes	Deng et al. (2018)
SB	Sodium butyrate (SB)	Immersed into different concentrations of SB solution	S. *aureus* and S. *aureus*	Plate counting and SPM osteomyelitis model in rat femur	Significantly inhibited bacterial proliferation	The increased phagocytic activities of macrophages and ROS production	Enhanced osteogenic differentiation of rBMSCs and reduced bone destruction and osteolysis *in vivo*	Yang et al. (2019)
Ag^+^/ZIF-8	Zn(NO_3_) _2_ AgNO_3_ 2-methylimidazole (Hmim)	*In situ* growth (SPEEK@ZIF-8) and then self-assembled Ag^+^ layer by layer	*E. coli* and S. *aureus*	Plate counting and FESEM	Extraordinary antibacterial ability	Steady release behavior of Ag^+^ and Zn^2+^	The cytotoxic effect of SPZA was slightly greater than that of SP due to the high concentration of bimetal ions	Yang et al. (2022)
MBD-14	MBD-14 lyophilized powder dissolved in phosphate-buffered solution	Dropped on hydrothermally treated SPEEK then lyophilized	S. *aureus*, *P. aeruginosa*, S. *aureus*, and *P. aeruginosa*	Plate counting and SEM osteomyelitis model in rat femur	Excellent antibacterial properties. Antibacterial effect increased with the concentration of MBD	MBD-14 long-term release	The expression of osteogenic differentiation-related genes and proteins was enhanced with increased MBD-14 concentration	Yuan et al. (2019)
Li^+^/AMP	LiOH- DOPA_4_-PEG_5_-RWRWRW peptide solution	Layer-by-layer on SPEEK (dip-coating)	*E. coli,* S. *aureus*, and S. *aureus*	Plate counting, SEM, and TEM osteomyelitis model in rat femur	Inhibited the bacteria adhesion and survival antibacterial efficiency reached 95.03%	AMP sequence, RWRWRW, can kill bacteria	Li^+^ promoted the osseointegration and osteogenic differentiation activity	Ning Li et al. (2021)
SA (CGA)/BFP	SA and CGA solution (4 mg/ml) grafted with BFP	Dip-coating then activated by EDC and NHS in MES buffer	*E. coli* and S. *aureus*	Plate counting and SEM	Noticeable antibacterial effect and inhibited the bacteria adhesion	CGA release	SA enhanced biocompatibility and BFP stimulated the proliferation and differentiation of osteoblasts	He et al. (2019)

However, sulfur may have negative effects on the human body, such as the synthesis of low-valent sulfurous compounds, to generate oxygen-free radicals, and damaged cells (Meng et al., 2005). Therefore, whether the corrosion and chemical composition changes of PEEK caused by concentrated sulfuric acid sulfonation can meet the safety requirements of long-term *in vivo* bone implants is worthy of further study. The balance of safety and antibacterial properties was explored by Ouyang et al. (2016). The research team washed SPEEK with water at different temperatures to obtain different residual sulfide concentrations on the surface. The higher the water temperature, the lower the residual sulfide concentration. *In vitro* experiments found that the antibacterial properties of SPEEK after treatment were not significantly reduced, especially the inhibitory effect on *Staphylococcus aureus*. The *in vivo* result was consistent with the *in vitro* experiments. In addition, the porous structure of SPEEK may have a profound impact on its biological properties. However, there is a lack of biomechanical research on bifunctional SPEEK in the aforementioned studies.

### Physical Modification

#### Surface Structure Modification

**FIGURE 4:**
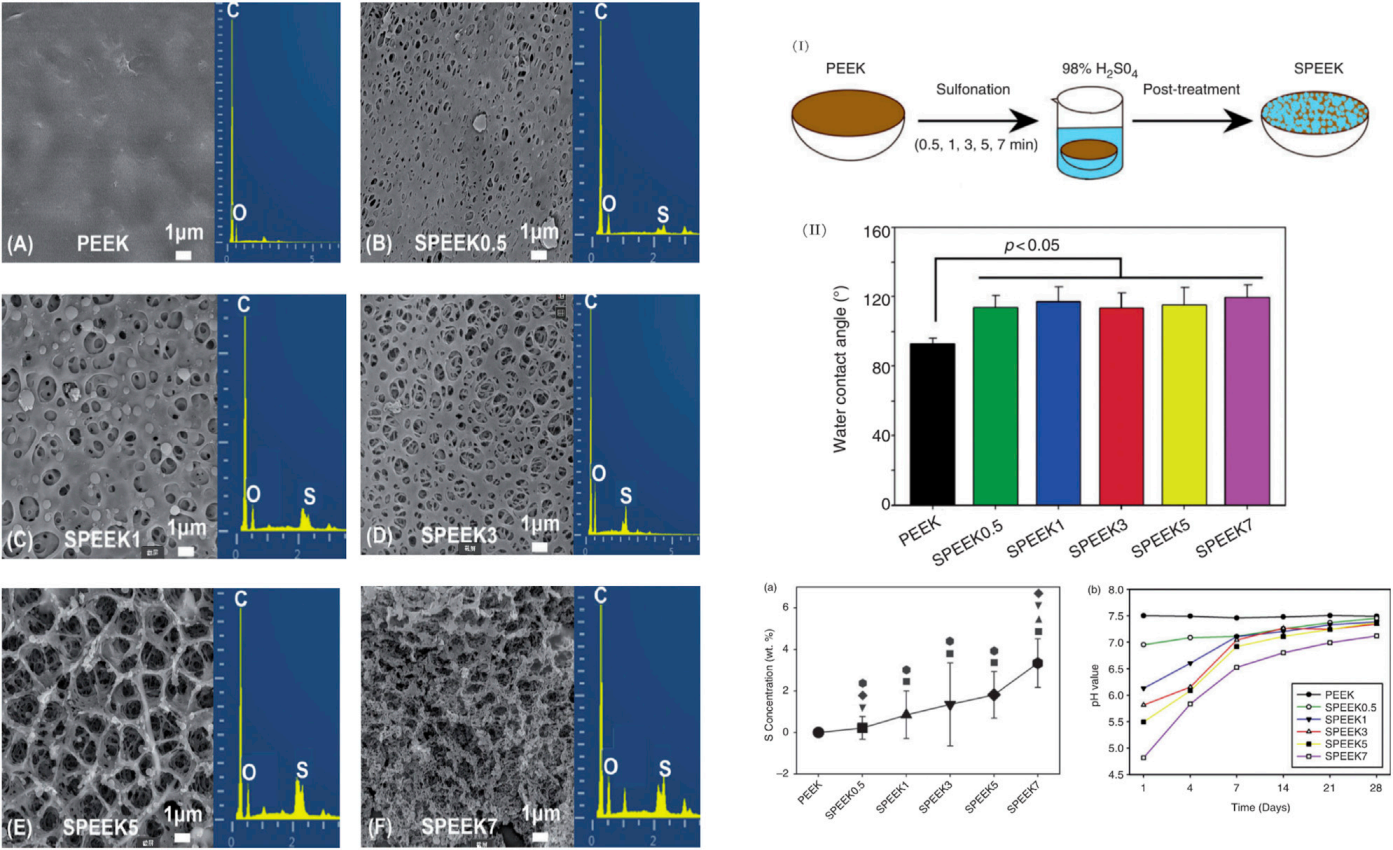
Sulfonation of PEEK. **(I)** Sulfonation times: 0.5 min (SPEEK0.5), 1 min (SPEEK1), 3 min (SPEEK3), 5 min (SPEEK5), and 7 min (SPEEK7); untreated PEEK as the control. **(II)** Quantitative comparison of water contact angles. (a) S concentration of each group measured by EDS (the marks represent *p* < 0.05); (b) pH values at 1, 4, 7, 14, 21, and 28 days. **(A–F)** Morphology of the SPEEK surface characterized by SEM (left row) and chemical composition characterized by EDS (right row). Reproduced from Ma R. et al (2020). Copyright (2020), with permission from SAGE Publications.

As mentioned in the introduction, the surface structure of PEEK affects cell adhesion and is prone to bacterial film formation. When bacteria attach to nanopillars smaller than their volume, the bacterial wall will rupture (Wang et al., 2017), so the formation of a micro-nano three-dimensional structure can enhance its biological activity and antibacterial properties. At present, there are many approaches to modify the surface structure of PEEK, such as UV irradiation, plasma treatment (Liu et al., 2021), fluorination (Chen et al., 2017), and nitration (Li et al., 2019), while all reported excellent antibacterial properties. The most widely used approach is sulfonation. Different sulfonation times can form different nano-network structures on the surface of PEEK, which can improve biocompatibility. But too long sulfonation time will lead to a disordered porous structure (Figure 4). The literature proved that sulfonation for 5 min was the ideal time to modify the surface structure of PEEK (Ma R. et al., 2020). In addition, based on sulfonation, other structural modification methods such as plasma shock can be combined to form three-dimensional micro-nanostructures with specific functional groups, which can further improve cell adhesion and antibacterial activity (Wang et al., 2018).

### Blend Injection Molding

Blending modification is to mix inorganic active substances into organisms to form biocomposites. This method has been proven by research studies to apply to the modification of bone endophytes and improve osteogenic activity and biocompatibility (Hajiali et al., 2017; Pang et al., 2019). Blend injection molding is characterized by improving the biomechanical properties of PEEK. As we know, the elastic modulus of pure PEEK (3.7–4.0 GPa) is lower than that of human cortical bone (7–30 GPa) (Ching et al., 2009). Tantalum is one of the metal materials that have been a hot topic studied in recent years. It has excellent histocompatibility and osseointegration, but the elastic modulus is too high. Scholars combined tantalum and PEEK into composite materials that proved to have good osseointegration and mechanical properties but with limited antibacterial property (Zhu et al., 2019; Hu et al., 2022). The antibacterial properties and osteogenic activity were further enhanced by further chelating the antibacterial compound genistein after sulfonation (Mei et al., 2021; Luo et al., 2022). Therefore, we believe that the relevant research on injection molding after particle blending should be tested to explore the changes in biomechanics.

At present, the antibacterial properties of blending modification have been extensively explored such as the common blending with antibacterial metal particles. PEEK and nano-zinc–magnesium silicate (nZMS) in different proportions have good antibacterial properties. When the nZMS content is 50%, the best antibacterial properties and mechanical properties are obtained (Tang et al., 2019). However, some scholars have found that metal particles affect histocompatibility or osseointegration performance after blending, so non-metallic particles are used. Silicon oxide and Si_3_N_4_ particles were incorporated into PEEK by melt blending, which had proved good antimicrobial properties with improved biocompatibility and osteogenic properties (Pezzotti et al., 2018; Hu et al., 2022). In addition to nanoparticles, antibacterial compounds can also be modified by blending: it was reported that chitosan, nanohydroxyapatite, and PEEK were made into mixed suspensions with different ratios, and all of them had good antibacterial effects on *S. aureus* and *E. coli*; moreover, chitosan could increase the stability of nanohydroxyapatite–PEEK binding (Abdulkareem et al., 2019). Blending modification still needs further research. In addition to the changes in biomechanical properties mentioned previously, most of the bioactive substances by blending modification are located in the matrix, which cannot be guaranteed with uniform and effective distribution, so the improvement of the surface properties is limited. For example, silver nanoparticles-coated PEEK has been shown to have antibacterial activity. However, the composite-mixed silver nanoparticles or silver ions with the PEEK powder through injection molding exhibited uneven particle distribution and failed to inhibit the bacteria adhering to the surface of the PEEK composite (Jaekel, 2012). To solve this problem, we can make improvements in two aspects. One is through the modification of the surface structure; the PEEK composite will form a micro-nanostructure on the surface, which can make the co-polymer-mixed particles obtain greater surface exposure, resulting in improved bioactivity and antimicrobial properties (Niu et al., 2020). Another approach is injection molding by 3D printing technology with a reasonable distribution of particles. It can be concluded that the modification of PEEK does not need to be limited to a single approach, and the synergy of two or even more modification methods can be explored, which provides more and better possibilities for the development of PEEK implant.

Some nanomaterials can generate heat under the irradiation of near-infrared light to inhibit bacterial growth. The blending modification of PEEK and photothermal materials can be applied to photothermal therapy. Most bacteria die at 55°C due to protein denaturation (Pan et al., 2016). At the same time, near-infrared rays can penetrate tissue well with high safety. Taking advantage of these properties, the research on antibacterial properties of nanomaterials with photothermal effects has grown exponentially in recent years, such as graphene composite nanomaterials and noble metal particles or compounds (Xu J.-W. et al., 2019; Song et al., 2020). Among them, graphene and its derived materials also have a good drug-controlled release effect (Mousavi et al., 2020). Photothermal materials have broad prospects in the field of biomedicine.

### 3D Printing Technology

**FIGURE 5:**
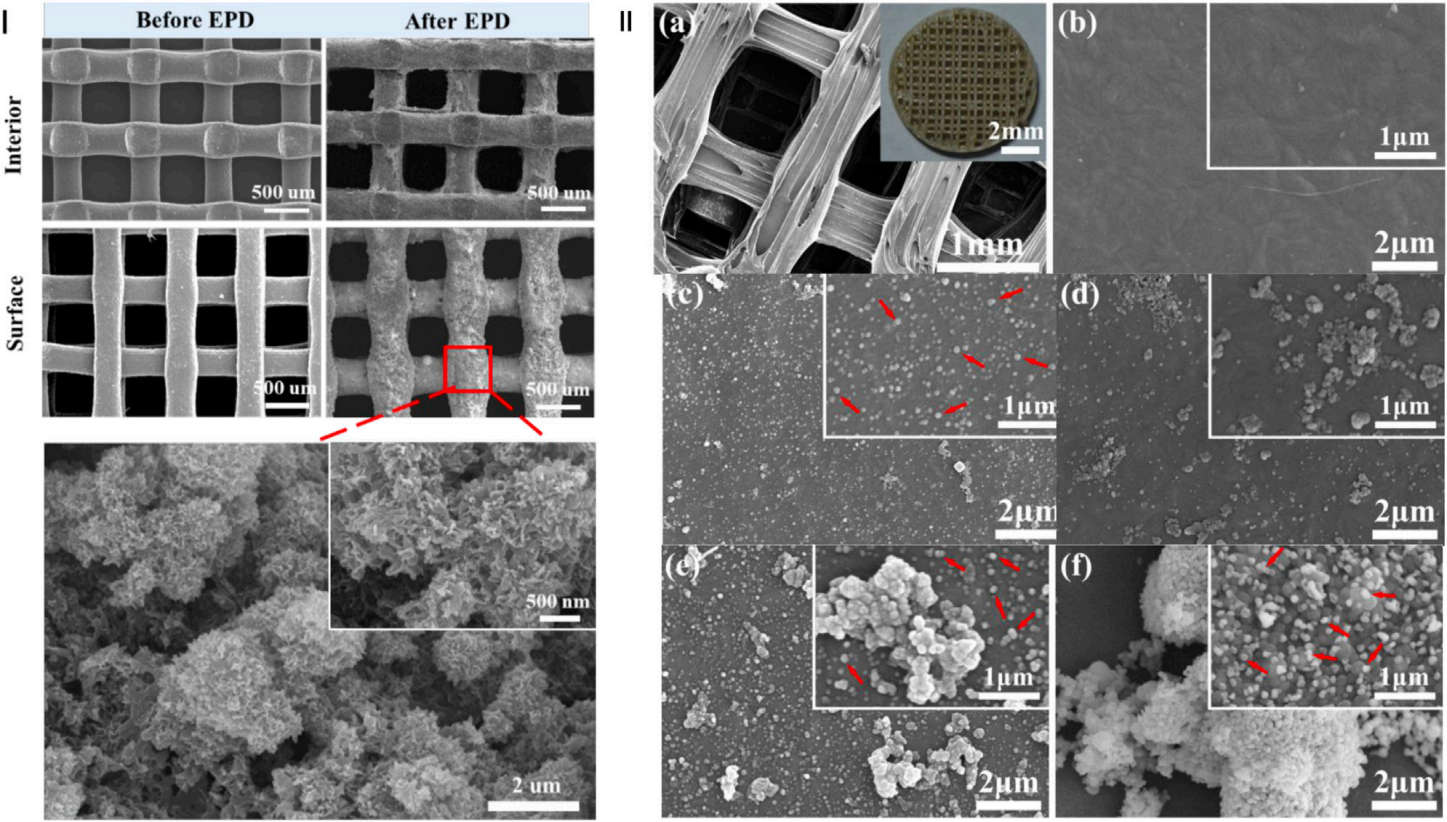
Modification of 3D-printed PEEK. **(I)** SEM images of the 3D-printed PEEK/graphene composite scaffold before and after drug-laden hydroxyapatite-coating deposition. Adapted with permission from Zhu et al (2021). Copyright (2021) American Chemical Society. **(II)** SEM overview of the 3D-printed PEEK scaffolds. **(A)** SEM zoom images, **(B)** 3D-printed PEEK, **(C)** AgNP-decorated PEEK, **(D)** apatite-decorated PEEK, **(E)** “PDA–Ag–PDA–apatite” multilayers on PEEK, and **(F)** “PDA–apatite–PDA–Ag” multilayers on PEEK. Red arrows point to AgNPs. Adapted with permission from Deng et al (2020). Copyright (2020) American Chemical Society.

At present, 3D-printed PEEK implants have entered medical application (Basgul et al., 2018; Atef et al., 2021), which have also been extended to composites mixed with other bioactive particles (Oladapo et al., 2020; Manzoor et al., 2021; Rodzeń et al., 2021b), which can achieve mechanical properties close to human cortical bone and good contact interface biological activity (Li S. et al., 2021; Rodzeń et al., 2021a). Some 3D-printed composite antibacterial scaffolds reported in the literature also demonstrated their potential for antibacterial modification of materials (Yang et al., 2018; Allen et al., 2020; Wang et al., 2021). This research is of great significance to the development of PEEK in the medical field. Oladapo et al (2021) elaborates on the scientific mechanism and some solutions for modifying PEEK by 3D printing. 3D printing technology can better control the distribution of each nanoparticle, solve the problem of insufficient exposure of antibacterial particles at the contact interface in blend injection molding to a certain extent, and also make the distribution of surface-modified particles more uniform and controllable, thereby improving biomechanics, surface properties, and antimicrobial activity of modified PEEK. Deng et al (2017) have produced 3D-printed PEEK decorated with nano-silver particles: *in vitro* experiments have shown that it has obvious inhibitory effects on Gram-positive bacteria (*S. aureus*) and Gram-negative bacteria (*E. coli*) and can destroy the biofilm formed After that, a “PDA–Ag–PDA” sandwich structure was further fabricated by 3D printing on a mesh-structured PEEK surface. *In vitro* experiments verified its intelligent-controlled release. When the pH decreased due to bacterial metabolism, Ag+ was released, and the experiments in mice also proved its good antibacterial properties and osseointegration (Deng et al., 2020). The 3D printing technique can also be used in photothermal therapy. It was reported in the literature that the antimicrobial drug-loaded hydroxyapatite (HA) coating was attached to the surface of a 3D-printed graphene/PEEK scaffold by electrophoretic deposition, and then the drug was released on-demand by near-infrared irradiation showing good bactericidal effect on *Escherichia coli* and MRSA (Zhu et al., 2021). At present, there are few reports on the antibacterial properties of 3D-printed PEEK. It may be because of the current shortcomings of 3D printing technology: first, its resolution is limited, the yield is low, and there are still difficulties and challenges in the manufacture of some sophisticated or complex structures (Tahayeri et al., 2018). Second, unlike sulfonation and other structural modifications to form a porous surface, 3D-printed PEEK forms a grid structure of both surface and matrix (Figure 5), so the mechanical property changes require further experimental research. However, it is believed that with the advancement of technology, especially the development of nanotechnology (Laird et al., 2021), 3D-printed PEEK must have broad prospects for the improvement of its osseointegration and antibacterial properties.

## Outlook

IAI is a *besorgniserregend* complication of orthopedic surgery with poor prognosis due to biofilm formation. The crucial factor is the initial bacteria adhesion onto implant interfaces. Therefore, it is very important to endow orthopedic implants with antibacterial properties. However, there are still a lot of experiments that need to be explored at present and in the future. First, the bacterial strains used in this literature are relatively single; *E. coli* and *S. aureus* are mostly used to represent Gram-negative and -positive bacteria. However, most implant infections in clinic are iatrogenic infections, the types of pathogens are diverse, and the incidence of drug-resistant bacteria is high. Therefore, the use of mixed pathogens in antibacterial experiments may better simulate implant infection than a single bacterial strain. Second, postoperative implant infections often occur within 4 weeks (Zhou et al., 2020) or even within 3 months (Borchardt and Tzizik, 2018). Considering the potential cytotoxicity and drug resistance risk of long-term antibacterial particles or compounds, the combination of chemical and physical modification should be explored more. The antibacterial particles or compounds on a physically modified PEEK provide a strong bactericidal effect in the early stage; then, the antibacterial structure alone can promote the attachment of surrounding tissues and the ability to resist the formation of biofilm achieving long-term antibacterial ability. Meanwhile, most of the surface chemical modifications of PEEK adopt conventional antibacterial metal particles or antibacterial drugs, while our group has reported the application potential of some non-antibacterial compounds in the treatment of IAI. Zhang et al (2020), Zhang S. et al (2021a), and Zhang S. et al (2021b) successively found and proved the antibacterial properties of flufenamic acid, diclofenac, and felodipine against implant biofilm and even drug-resistant bacteria such as MRSA infection. Finally, orthopedic implants not only need to resist long-term chemical erosion *in vivo* but also need to bear a variety of physical stress, such as friction, anti-fatigue, shear force, and violence due to different injury mechanisms. So, there is still a large amount of research work to be completed for the successful clinical transformation of antibacterial biofunctionalized PEEK: the stability of the coating under different stresses; whether the antibacterial substances could be released evenly and effectively; the granular reaction *in vivo*, and so on. The biomechanical examination also needs to be repeated after long-term implantation *in vivo*, and the detection of residual coating and the analysis of tissue around the implant are worthy of further exploration. In addition, with the improvement of 3D printing accuracy in the future, it will have a revolutionary impact on implant materials such as PEEK.

## Conclusion

PEEK is the most exciting orthopedic implant material in the past 20 years. A large number of research studies have investigated the biofunctionalization of PEEK with antibacterial. Good antibacterial properties are not only one of the key elements for the success of implants but also can be used to treat difficult clinical diseases such as infection and osteomyelitis. This review summarized the research results of many scholars in recent years and analyzed the approaches to biofunctionalize PEEK for antibacterial and specific methods of modification and then looked forward to the future research direction and problems to be further solved, contributing to the development of the antibacterial scheme of polyetheretherketone.
